# Selected Cytokines and Matrix Metalloproteinases in Pathogenesis and Treatment of Multiple Sclerosis

**DOI:** 10.3390/ijms27188079

**Published:** 2026-09-11

**Authors:** Jan Mroczko, Adrianna Romanowicz, Marta Łukaszewicz-Zając, Barbara Mroczko, Agnieszka Kulczyńska-Przybik, Lidia Glodzik

**Affiliations:** 1 Department of Neurodegeneration Diagnostics, Medical University of Bialystok, 15-276 Bialystok, Poland; mjanek2003@gmail.com (J.M.); adrianna.romanowicz@sd.umb.edu.pl (A.R.); agnieszka.kulczynska-przybik@umb.edu.pl (A.K.-P.); 2 Department of Biochemical Diagnostics, University Clinical Hospital of Bialystok, 15-276 Bialystok, Poland; marta.lukaszewicz-zajac@umb.edu.pl; 3 Department of Biochemical Diagnostics, Medical University of Bialystok, 15-276 Bialystok, Poland; 4 Brain Health Imaging Institute, Department of Radiology, Weill Cornell Medicine, New York, NY 10021, USA; lig4005@med.cornell.edu

**Keywords:** multiple sclerosis, MMPs, interleukins

## Abstract

Multiple sclerosis (MS) is a progressive autoimmune disease of the nervous system (NS). Axonal degeneration, demyelination, and inflammation are the main mechanisms leading to the development of this disorder. In the course of MS, parenchymal cells in the NS are activated to produce cytokines and cytokine-induced proteins, including matrix metalloproteinases (MMPs). The presence of these enzymes and cytokines in the central nervous system (CNS) during an inflammatory response may suggest their involvement in the pathogenesis or progression of MS. A precise determination of the role of cytokines and MMPs in the development of MS may contribute to a better understanding of the causes of this disease and to preventing its progression. Therefore, this study was conducted to determine the role of selected cytokines and MMPs in the pathogenesis of MS and its potential treatments. It was shown that specific cytokines and MMPs act as critical pathophysiological drivers and biomarkers in MS. While acute neuroinflammation and blood–brain barrier disruption are mediated by interleukin (IL)-3, IL-5, IL-6, and MMP-9, progressive neurodegeneration correlates with MMP-3 and constitutive MMP-2. In conclusion, MMP-1 serves as a diagnostic biomarker that differentiates early MS from clinically isolated syndrome, whereas IL-9, IL-10, and IL-13 represent key immunomodulatory targets for neuroprotective strategies.

## 1. Introduction

Multiple sclerosis (MS) is classified as a neurodegenerative disease with a distinct inflammatory component [1]. The main characteristic of the disease is moderate axonal preservation with demyelinated areas. There are four main types of MS: relapsing–remitting MS (RRMS) with periods of remission and relapse; secondary progressive MS (SPMS) characterized by deterioration of symptoms; primary progressive MS (PPMS) without relapses or remissions; and progressive relapsing MS (PRMS), which is the rarest form, with occasional relapses without remission. Unlike most neurodegenerative diseases, which occur in older age, multiple sclerosis affects younger people. MS can occur in individuals between 20 and 45 years of age and can lead to disability [2,3]. Inflammation-induced neurodegeneration is a defining feature of multiple sclerosis, but its underlying mechanisms remain unclear. Based on recent research, the immunological process of MS relates to its conversion to SPMS and forms the basis for disease-modifying therapies (DMTs) [4,5]. Accumulating evidence suggests that the clinical course of multiple sclerosis might be considered a continuum, from predominantly localized, acute injury to widespread inflammation and neurodegeneration [6]. Some investigations support the concept of compartmentalized B-cell inflammation in progressive MS, which might be connected to an imbalance between matrix metalloproteinases (MMPs) and their tissue inhibitors (TIMPs), as well as with activation of selected cytokines [7,8,9]. Absinta et al. defined ‘microglia inflamed in MS’ (MIMS) and ‘astrocytes inflamed in MS’, glial phenotypes that demonstrate neurodegenerative programming. The MIMS transcriptional profile overlaps with that of microglia in other neurodegenerative diseases [10]. Additionally, the presence of MMPs increased microglial abundance, highlighting their indirect role in central nervous system (CNS) demyelination [11].

MMPs regulate several processes, including inflammation, microglial activation, and blood–brain barrier (BBB) disruption. MMP activity leads to an increase in membrane permeability by altering the extracellular matrix (ECM) and tight junctional features [12]. MMPs are also part of the neuroinflammatory response during ischemic injury, infection, and vascular dementias [13]. MMPs can be divided into four subgroups: Stromelysins (MMP-11, -10, -7, and -3), Collagenases (MMP-1, -8, and -13), Gelatinases (MMP-2 and -9), and Membrane type (MT)-MMPs. Stromelysins can degrade the ECM [14,15,16,17]. Gelatinases are involved in neurogenesis and angiogenesis, and MT-MMPs activate developmental components and proteases at the cell surface. Some studies have shown that MMPs are involved in vascular cognitive impairment (VCI)-associated neurodegeneration [13].

Numerous investigations have revealed close interplay between MMPs and interleukins in neuroinflammation and neurodegeneration. Interleukins not only regulate MMP expression and activity but are themselves influenced by MMP-mediated mechanisms. Cytokines are small signaling proteins secreted by immune, stromal, and tumor cells that regulate cellular communication by binding to specific receptors on target cells. Through autocrine, paracrine, or endocrine signaling, they control processes such as cell proliferation, differentiation, and immune responses. Cytokines are broadly classified into interleukins (ILs), interferons (IFNs), tumor necrosis factors (TNFs), colony-stimulating factors (CSFs), chemokines, and members of the transforming growth factor-β (TGF-β) superfamily [18,19]. Cytokines, especially interleukins, play a central role in the inflammatory processes underlying MS. An imbalance between pro- and anti-inflammatory cytokines, particularly those produced by T helper 1 (Th1) and T helper 2 (Th2) cells, together with the involvement of other T-helper cell subsets, is considered a major contributor to MS development [20,21].

Therefore, the aim of our review was to summarize recent advances in research on the role of selected cytokines, particularly ILs, and MMPs in the immunopathogenesis of MS and to highlight their potential implications for the development of innovative therapeutic approaches targeting these proteins. Stromelysins (MMP-3, MMP-7) have been selected as the main factors involved in the degradation of ECM as well as microglial activation (MMP-3) and cytokine release, while Collagenase (MMP-1) has been chosen due to its important role in the cleavage of fibrillar collagen type I and in differentiation between early MS and clinically isolated syndrome (CIS), as well as because it was found to correlate with lesion volume and gadolinium-enhancing lesions on magnetic resonance imaging (MRI). Moreover, Gelatinases (MMP-2 and MMP-9) are important supporters of neurogenesis and angiogenesis, while MMP-14, as a membrane-type (MT) MMP, plays a pivotal role in structural and signaling interactions between neurons, glial cells, and the ECM. The cytokines were selected based on recent evidence supporting their involvement in MS pathophysiology, disease activity, progression, or potential clinical relevance. Therefore, cytokines representing both pro-inflammatory and anti-inflammatory mechanisms involved in MS, thereby reflecting the complex balance between immune activation and neuroinflammation as well as immunoregulatory and neuroprotective processes, were included in the paper.

This review was based on a literature search conducted primarily in PubMed and Google Scholar. The search focused on using combinations of terms such as “multiple sclerosis”, “cytokines”, “interleukins”, “matrix metalloproteinases”, “inflammation”, and the names of individual molecules discussed in this review. Attention was given to original studies evaluating cytokine concentrations in biological samples obtained from patients with MS, primarily serum and cerebrospinal fluid. Studies investigating their expression in CNS tissues, including brain tissue and demyelinating lesions, as well as in immune cells, were also considered. Experimental studies, including in vitro investigations and experimental autoimmune encephalomyelitis (EAE) models, were added when they provided relevant mechanistic or therapeutic insights. Priority was given to recent original studies to provide an up-to-date overview of current knowledge, while selected older studies were included when relevant to the biological or pathophysiological context.

Finally, this manuscript, as a narrative review, has been created based on current literature presenting data on the potential role of the above-mentioned proteins in the pathophysiology of MS as well as in the treatment of this disease.

## 2. The Significance of Selected Cytokines in Multiple Sclerosis

### 2.1. Interleukin-6

Interleukin-6 (IL-6) is a pleiotropic cytokine involved in the regulation of immune responses and the induction of the acute-phase response [22]. It is produced by both immune cells, including T and B lymphocytes, macrophages, microglia, and non-immune cells such as endothelial cells and neurons. IL-6 signaling occurs via two pathways: classical signaling through the membrane-bound IL-6 receptor (IL-6R) and trans-signaling mediated by the soluble IL-6 receptor (sIL-6R), which interacts with the ubiquitously expressed glycoprotein 130 (gp130). While classical IL-6 signaling is considered to exert predominantly anti-inflammatory effects, trans-signaling is responsible for the pro-inflammatory actions of IL-6. Furthermore, in the presence of TGF-β, IL-6 promotes the differentiation of naïve CD4^+^ T cells into Th17 cells while inhibiting the generation of regulatory T cells (Tregs). Increased IL-6 has been detected in active demyelinating lesions of patients with MS [23]. A study by Itorralba et al. demonstrated that elevated cerebrospinal fluid (CSF) IL-6 concentrations were observed in patients with progressive MS and were associated with higher Expanded Disability Status Scale (EDSS) and Multiple Sclerosis Severity Score (MSSS) values, indicating a relationship between IL-6 and clinical disability [24]. Furthermore, the positive correlation between IL-6 and CSF glial fibrillary acidic protein (GFAP) levels suggests that IL-6 may be linked to ongoing astroglial activation and chronic intrathecal inflammation, supporting its potential involvement in the progression of MS [24]. Another study investigating the role of IL-6 in MS demonstrated that IL-6 was detected significantly more frequently in the CSF of patients with CIS, RRMS, and SPMS than in healthy individuals. Similar IL-6 detectability across different disease phenotypes suggests that IL-6-mediated signaling is not restricted to a specific clinical form of MS but may be involved in shared pathogenic mechanisms throughout the course of the disease [25]. The involvement of IL-6 in MS-related inflammation was further supported by another study showing a positive correlation between CSF IL-6 concentrations and the number of contrast-enhancing lesions in the thoracic spinal cord, onMRI, in patients with newly diagnosed MS. These findings suggest that IL-6 CSF levels may serve as an indicator of disease activity and reflect the extent of ongoing neuroinflammation [26,27]. The relationship between IL-6 and vitamin D status in MS has also been investigated in several studies. Another study reported a negative correlation between CSF IL-6 concentrations and serum vitamin D levels in patients with MS, suggesting that reduced vitamin D availability may be associated with enhanced inflammatory activity, as reflected by increased IL-6 production in the CNS [27,28]. Consistently, other studies showed that lower vitamin D levels in MS patients are associated with increased disease activity and faster progression [29]. These observations emphasize the relevance of IL-6 as an indicator of inflammatory activity in MS and its link to vitamin D status, which influences the immune response by modulating inflammatory processes associated with the disease.

### 2.2. Interleukin-9

**Figure 1:**
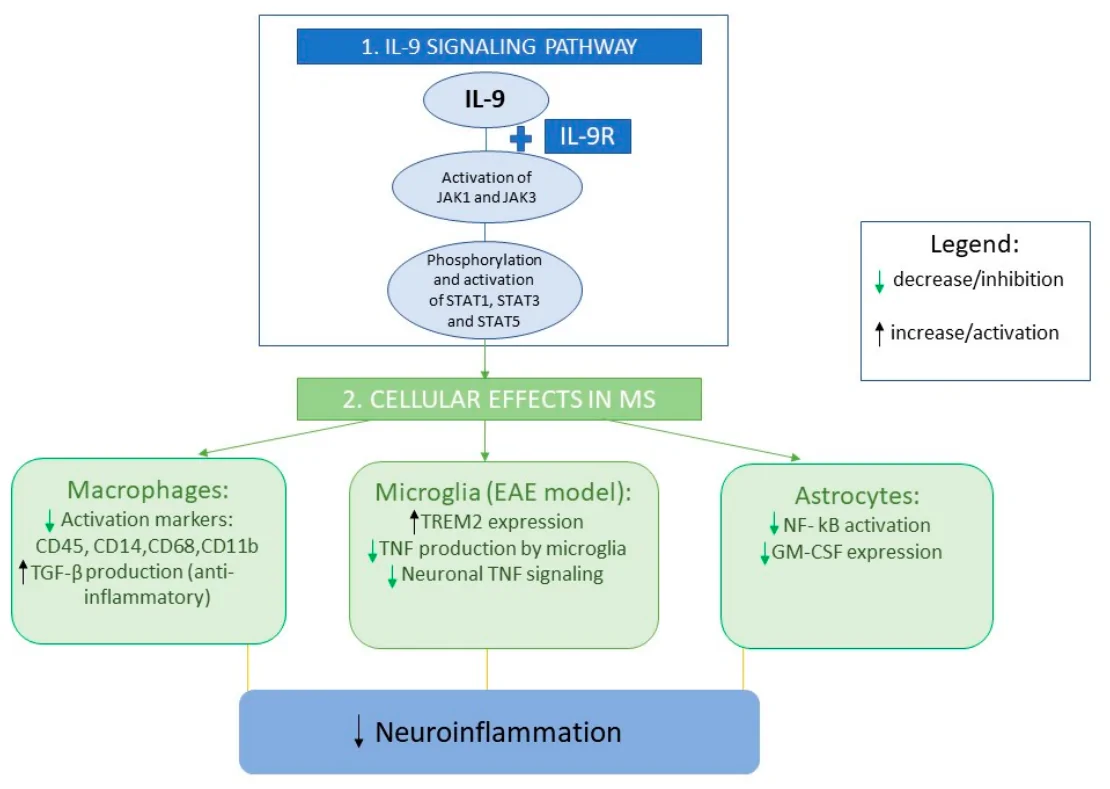
The role of interleukin-9 (IL-9) in regulating neuroinflammation in multiple sclerosis [30,31,32,33,34,35,36].

Interleukin-9 (IL-9) exerts its biological effects through the interleukin-9 receptor (IL-9R), a heterodimer composed of a specific α-chain and the common γ-chain shared with the IL-2 receptor. Binding of IL-9 to IL-9R activates Janus kinase (JAK)1 and JAK3, leading to phosphorylation and activation of signal transducer and activator of transcription (STAT)1, STAT3, and STAT5 signaling pathways [30,31]. Although the downstream effects of IL-9 have been primarily characterized in mast cells during allergic responses, accumulating evidence suggests that IL-9 also participates in the regulation of autoimmune inflammation, including in MS. A study by Donninelli et al. demonstrated that the highest expression of this cytokine in the CNS was observed in macrophages, microglia, and CD4^+^ T lymphocytes. Monocytes/macrophages were identified as the circulating immune cells most responsive to IL-9, whereas IL-9R expression on macrophages/microglia was confirmed in post-mortem brain tissue from MS patients. Functional analyses further showed that IL-9 activated STAT1, STAT3, and STAT5 signaling, while attenuating the inflammatory phenotype of macrophages by reducing the expression of activation markers, including cluster of differentiation (CD) 45, CD14, CD68, and CD11b. In parallel, IL-9 enhanced the production of the anti-inflammatory cytokine -TGF-β, supporting a potential immunoregulatory role for IL-9 in the inflammatory environment of MS [32]. Ruocco et al. reported an inverse correlation between CSF IL-9 levels and disease course in patients with RRMS [33]. Similarly, Matsushita et al. observed reduced CSF IL-9 concentrations during clinical relapses, whereas IL-9 levels increased following prednisolone treatment [34]. Collectively, these observations suggest that decreased IL-9 levels may be associated with enhanced inflammatory and neurodegenerative processes in patients with de novo RRMS. Therefore, CSF IL-9 may represent a potential prognostic biomarker for monitoring disease progression and evaluating therapeutic response [33,34]. A study using the experimental autoimmune encephalomyelitis (EAE) model supported the protective role of IL-9 in MS. Both the systemic and local administration of IL-9 reduced clinical disability, neuroinflammation, and synaptic damage. These effects were linked to enhanced receptor expressed on myeloid cells-2 (TREM2), decreased TNF production by microglia, and the suppression of neuronal TNF signaling. Overall, IL-9 may exert both immunomodulatory and neuroprotective effects, highlighting its potential as a therapeutic target in MS [35]. Another study demonstrated that IL-9 promotes the transition of astrocytes from a pro-inflammatory to an anti-inflammatory phenotype by inhibiting NF-κB activation. At the same time, IL-9 reduced the expression of granulocyte-macrophage colony-stimulating factor (GM-CSF), a cytokine that plays a key role in microglial activation and the maintenance of inflammation within the CNS. These findings indicate that IL-9 may regulate the astrocyte–microglia axis, thereby contributing to the attenuation of neuroinflammation and highlighting its potential therapeutic relevance in MS, as shown in Figure 1 [36].

### 2.3. Interleukin-10

Interleukin-10 (IL-10) is one of the principal anti-inflammatory cytokines involved in regulating immune responses. It exerts potent immunosuppressive effects, primarily by inhibiting the activation and function of antigen-presenting cells, and is predominantly produced by activated leukocytes under inflammatory conditions [37]. Recent findings indicate that the role of IL-10 in MS is complex and may vary depending on disease stage and the biological material analyzed. Elevated serum IL-10 levels have been reported in patients with MS compared with healthy individuals and were associated with a more favorable clinical profile. Higher IL-10 concentrations were linked to lower disease burden, including reduced disability, fewer relapses, shorter disease duration, better physical performance, and less severe depressive symptoms. Furthermore, increased IL-10 levels were associated with better quality of life [38]. However, recent studies have reported different findings. Mado et al. observed significantly lower serum IL-10 concentrations in patients with MS compared with healthy controls, while within the MS group, the highest IL-10 levels were found in patients with greater disability (EDSS ≥ 4.0) [39]. In addition, Gębka-Kępińska et al. reported lower CSF IL-10 concentrations in RRMS patients compared with controls, with IL-10 levels decreasing over time from symptom onset [27]. These findings indicate that the relationship between IL-10 concentrations and MS severity is not straightforward and may depend on disease stage, MS phenotype, treatment status, and the biological material analyzed. Evidence from CSF analyses indicates that IL-10 concentrations were positively correlated with changes in EDSS scores during the first year after MS diagnosis. In addition, a positive correlation was observed between CSF IL-10 concentrations and the volume of subcortical brain structures, including the caudate nucleus. Furthermore, the concurrent upregulation of IL-10 and IL-1β in the CSF of patients with MS and in the striatum of EAE mice suggests a potential antagonistic interaction between these cytokines. Mechanistically, IL-10 reduced glutamatergic neurotransmission, enhanced GABAergic transmission, and reversed IL-1β-induced synaptic abnormalities in the EAE model. Overall, IL-10 may contribute to neuroprotection in MS through the modulation of synaptic transmission and attenuation of IL-1β-driven inflammatory synaptopathy. These observations support further investigation of IL-10 as a potential therapeutic target aimed at limiting neurodegeneration in MS [40]. Interestingly, prior Epstein–Barr (EBV) infection has been associated with an increased risk of multiple sclerosis, potentially through IL-10-related mechanisms. The accumulation of latently EBV-infected B cells in the CNS, in combination with the activity of cellular IL-10 and EBV-encoded IL-10, may contribute to the dysregulation of T cell responses and the establishment of a pro-inflammatory environment that facilitates MS pathogenesis [41]. In another study, researchers revealed that regulatory B10 (a special subset of regulatory B cells with immunoregulatory function that is fully attributed to IL-10) cells, through their ability to produce IL-10, contribute to recovery from EAE in animal models, suggesting their potential therapeutic relevance in MS. Although direct administration of IL-10 appears promising, its clinical application is limited by its short half-life and restricted biological activity. Therefore, strategies aimed at enhancing or restoring the function of IL-10-producing B10 cells may represent a more effective approach. Importantly, the immunosuppressive activity of B10 cells is tightly regulated and depends on antigen-specific interactions and the local immune microenvironment, allowing for targeted modulation of immune responses without inducing generalized immunosuppression. Consequently, in vivo manipulation or ex vivo expansion of B10 cells has emerged as a promising therapeutic strategy for MS [42].

### 2.4. Interleukin-13

Interleukin-13 (IL-13), produced predominantly by Th2 lymphocytes, is a pleiotropic cytokine with well-established anti-inflammatory properties [43]. Although IL-13 is generally recognized as an anti-inflammatory cytokine, its role in MS appears to be more complex. An increased proportion of IL-13-producing CD4^+^ T cells in the CFS was observed in patients with RRMS during relapse compared with those in remission. Furthermore, the frequency of IL-13-producing T cells positively correlated with EDSS scores. IL-13-producing T cells were also detected in the meninges and active spinal cord lesions of patients with MS, suggesting that IL-13 may participate in local immune responses within the CNS [44]. Evaluation of serum IL-13 levels showed that patients with RRMS exhibited significantly reduced concentrations of this cytokine compared with healthy controls, which suggests that reduced IL-13 production contributes to disease pathogenesis. Furthermore, serum IL-13 levels were negatively correlated with EDSS scores, indicating an association between lower IL-13 concentrations and greater neurological disability. In contrast, no significant associations were observed between IL-13 levels and MRI lesion burden, relapse frequency, oligoclonal band positivity, immunoglobulin G (IgG) index, or treatment with fingolimod or glatiramer acetate [45]. A recent multicenter study by Rodero-Romero et al. also demonstrated significantly lower serum IL-13 levels in all analyzed groups of patients with MS compared with healthy controls. Together with reduced IL-4 levels, these findings were suggested to indicate inhibition of the Th2 response in MS [46]. Genetic studies have also suggested a role for IL-13 in susceptibility to MS. The rs20541 (R130Q) polymorphism of the *IL13* gene was significantly associated with an increased risk of MS, with A allele carriers exhibiting higher susceptibility than GG homozygotes. However, this polymorphism was not associated with clinical characteristics of the disease, including age at disease onset or EDSS score [47]. A study by Azimzadeh et al. examined the immunoregulatory and neuroprotective effects of human adipose tissue-derived stem cells (hADSCs) overexpressing IL-11 and IL-13 in mice with EAE. In this model, transplantation of hADSCs engineered to express IL-11 and IL-13 attenuated disease severity, reduced inflammatory cell infiltration into the spinal cord, and promoted remyelination and oligodendrogenesis. These findings suggest that enhancing IL-13 expression may augment the immunomodulatory and neuroprotective effects of stem cell-based therapies for MS [48].

### 2.5. Other Interleukins

Interleukin-3 (IL-3) is a pleiotropic cytokine and hematopoietic growth factor belonging to the colony-stimulating factor family. It has been implicated in the regulation of immune responses and is involved in the pathogenesis of various inflammatory and autoimmune diseases [49]. A study conducted by Kiss et al. demonstrated that astrocytes and infiltrating CD4^+^ T cells are the primary sources of IL-3 within the inflamed CNS in MS. IL-3 acts on IL-3Rα-expressing myeloid cells, promoting their chemotactic and migratory properties, which enhances the recruitment of immune cells into the CNS and amplifies the inflammatory response. Furthermore, the presence of IL-3 in the CFS was associated with both MS diagnosis and disease severity. Collectively, these findings suggest that the IL-3–IL-3Rα axis plays an important role in MS pathogenesis by promoting neuroinflammation through bidirectional communication between immune cells and resident glial cells [50].

Interleukin-5 (IL-5) is an anti-inflammatory cytokine primarily involved in eosinophil growth, differentiation, and activation during allergic responses. In MS, IL-5 appears to contribute to immune regulation by enhancing anti-inflammatory mechanisms and supporting regulatory T cell responses [51]. A study investigating the rs2069812 polymorphism of the *IL5* gene demonstrated that the T allele was associated with reduced IL-5 expression in the CSF of patients with RRMS and with a more pro-inflammatory cytokine profile. In patients with progressive MS, the T allele was likewise associated with lower IL-5 concentrations. Moreover, in RRMS patients, No Evidence of Disease Activity (NEDA-3) at three years of follow-up was detected. These findings suggest that genetic variation within IL-5 may influence the inflammatory milieu in the CNS and contribute to MS pathogenesis [52]. Interleukin-27 (IL-27) may be primarily associated with the early phase of MS, as higher serum concentrations were observed in RRMS patients with an earlier disease onset. In RRMS patients, serum interleukin-23 (IL-23) concentrations were significantly higher among individuals carrying the GG genotype of the R381Q polymorphism compared with AG genotype carriers. No significant associations were found between serum IL-27 or IL-23 concentrations and disease duration or relapse frequency. However, IL-23 concentrations were positively correlated with the duration of disease-modifying treatment, including glatiramer acetate and interferon beta therapy, in patients with SPMS [53].

Cytokines are involved not only in MS pathogenesis but may also be associated with the response to disease-modifying therapies. Therefore, several studies have investigated changes in cytokine levels during MS treatment and their potential relationship with clinical disease activity. The effect of MS treatment on cytokine profiles has been investigated in patients receiving natalizumab. After eight months of treatment, significant reductions in serum IL-17F and IL-31 levels were observed in patients with RRMS. Moreover, higher IL-17F, IL-1β, and IL-31 concentrations were positively correlated with the number of relapses, while IL-1β and TNF-α levels were positively associated with EDSS scores. IL-17F and IL-31 were also proposed as potential biomarkers for assessing the effectiveness of natalizumab treatment [54]. Ocrelizumab treatment has also been associated with changes in TNF-related immune responses. In patients with PPMS, six months of treatment significantly reduced the number of B cells producing the pro-inflammatory cytokines TNF-α, IL-6, and GM-CSF [55]. Moreover, a recent study showed that higher baseline TNF messenger ribonucleic acid (mRNA) expression in peripheral blood mononuclear cells (PBMCs) was associated with an increased risk of disease activity and a greater likelihood of disability progression during 24 months of ocrelizumab treatment, suggesting its potential value as a prognostic marker of treatment response [56]. In addition, IL-17-producing CD8+ T (Tc17) cells are enriched in active MS lesions, suggesting their involvement in disease pathogenesis. Dimethyl fumarate treatment (DMF) reduced the frequency of Tc17 cells, but not Th17 cells. Importantly, a significant reduction in Tc17 cells was observed in patients who achieved No Evidence of Disease Activity-3 (NEDA-3) after approximately one year of treatment, whereas no significant change was found in non-responders. These findings suggest that suppression of Tc17 responses may be associated with a favorable response to DMF treatment [57]. The effects of another MS treatment, cladribine, were investigated in PBMCs obtained from untreated patients with RRMS. Exposure to cladribine increased IL-4 and decreased TNF-α secretion, indicating a shift toward a more anti-inflammatory cytokine profile. Decreased IFN-γ secretion was also observed in patients with shorter disease duration, whereas no significant changes were found for IL-17 [58].

Collectively, the studies discussed above highlight the significant clinical relevance of the selected interleukins in multiple sclerosis, emphasizing their involvement in disease pathogenesis, inflammatory activity, and clinical course. Moreover, some of these interleukins are potential therapeutic targets. A summary of the main findings concerning selected cytokines is presented in Table 1.

## 3. The Significance of Selected Matrix Metalloproteinases in Multiple Sclerosis

### 3.1. Matrix Metalloproteinase-3

Numerous studies have indicated that matrix metalloproteinase-3 (MMP-3) is involved in neurodegeneration and neuroinflammation. Serine proteinases are responsible for the extracellular activation of proMMP-3 [59]. MMP-3 cleaves components of the ECM, such as aggrecan, fibronectin, laminin, and tenascins, as well as TNF-α and interleukin-1β [60]. MMP-3 acts as a signaling molecule in apoptosis and is extracellularly released, leading to the activation of microglia, which then produce various cytotoxic proinflammatory molecules.

**Figure 2:**
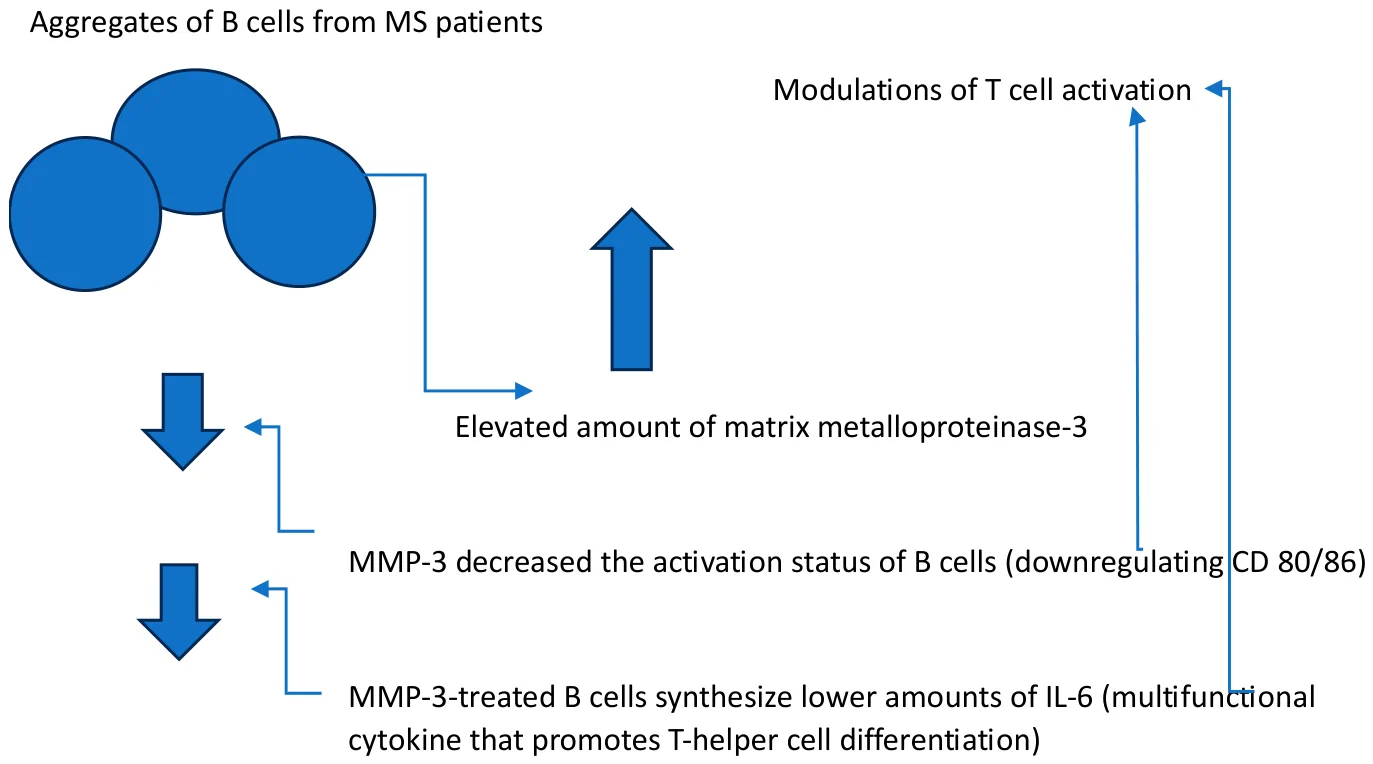
Therapeutic potential of the interaction between matrix metalloproteinase-3 (MMP-3) and B cells [7,61,62,63,64,65,66,67,68,69].

Chunder et al. studied MMP-3’s role in the modulation of B cell responses in MS. Autopsied brain sections from multiple sclerosis patients containing aggregates of B cells contained a significantly higher quantity of matrix metalloproteinase-3 compared to subjects without MS [7]. In vitro experiments demonstrated that MMP-3 decreased the overall activation status of B cells by downregulating CD69, CD80, and CD86, playing a critical role in the modulation of T cell activation. Furthermore, matrix metalloproteinase-3-treated B cells produced significantly lower amounts of interleukin-6. In MS, memory B cells are the main pathogenic B cell subtype [61,62,63]. Both CD80 and CD86 are over-expressed on B cells in patients with MS [64,65]. MMP-3 decreased the overall activation status of B cells by modulating the costimulatory molecules CD80/CD86. Therefore, MMP-3 significantly decreased the production of IL-6, which is a proinflammatory multifunctional cytokine that promotes T-helper cell differentiation [66,67]. The therapeutic significance of the neutralizing effects of IL-6 has been indicated for several autoimmune diseases [68] and might be relevant to MS treatment [69], as shown in Figure 2. However, the proposed MMP-3-B-cell-IL-6 therapeutic axis has been presented only as a hypothesis.

MMP-3 also affects B cells at the mRNA level. CD40, a co-stimulatory receptor protein [70], was significantly downregulated in B cells treated with MMP-3.

Furthermore, the findings of Moccia et al. support the concept of targeting compartmentalized B-cell inflammation in progressive MS [8]. They investigated the clinical and pathologic correlations of compartmentalized perivascular B cells in postmortem progressive MS brains. Brain slices were acquired from 11 people with SPMS, 5 people with PPMS, and 4 subjects without MS and immunostained for B lymphocytes (CD20), T lymphocytes (CD3), cytotoxic T lymphocytes (CD8), neuronal neurofilaments (NF200), myelin (SMI94), macrophages/microglia (CD68 and IBA1), astrocytes (GFAP), and mitochondria (voltage-dependent anion channel and cytochrome c oxidase subunit 4). The CD20 immunostaining intensity was higher in PPMS patients compared with controls. Moreover, higher CD20 immunostaining intensity was associated with younger age at onset, wheelchair dependence, and elevated risk of death. Interestingly, the presence of perivascular B cells correlated with worse clinical outcomes and CNS-compartmentalized inflammation. These findings may support the concept of targeting compartmentalized B-cell inflammation in progressive MS through the potential use of MMP-3 in the treatment of MS. Importantly, interactions between extracellular matrix structure and astrocytes can contribute to neuroinflammation. At the synaptic level, astrocytes effectuate not only neurotransmitter regulation but also synaptic formation, maturation, pruning, and stability [71]. Based on the abovementioned findings, it can be concluded that MMP-3 plays an important role in neuroinflammation and neurodegeneration in the course of MS and may be therapeutically targeted, especially by significantly decreasing the production of IL-6, which is a proinflammatory multifunctional cytokine.

### 3.2. Matrix Metalloproteinase-9 and Matrix Metalloproteinase-2

InMS, MMP activity in tissues is the result of a balance between MMPs and their TIMPs [72]. Matrix Metalloproteinase-9 (MMP-9) predominates in acute MS lesions and is inhibited by its tissue inhibitor—TIMP-1. It was shown that the MMP-9/TIMP-1 ratio in serum was elevated in active MS patients (defined either clinically or by magnetic resonance imaging) compared to those with inactive disease [72]. However, the CSF MMP-9/TIMP-1 ratio did not differ between MS subtypes, indicating systemic rather than CNS-restricted changes [72]. The authors concluded that high MMP-9 activity characterizes short-duration relapsing and active forms of the disease [72]. In a recent study, Bernaerst et al. investigated the role of MMP-9 in demyelination and remyelination, using two complementary models: lysophosphatidylcholine (LPC)-induced demyelination in ex vivo brain slices and cuprizone (CPZ)-induced demyelination in vivo [11]. In the LPC-treated slices, they found that MMP-9 deficiency impaired remyelination via microglia/macrophage-mediated mechanisms; however, MMP-9 deficiency had minimal impact on demyelination or remyelination in the CPZ model [11]. These findings demonstrated that MMP-9 did not directly regulate demyelination and remyelination in the CPZ-induced demyelination model, but increased microglial abundance, highlighting its indirect role in CNS demyelination [11]. In a different study, Fainardi et al. suggested that a shift in the MMP-9/TIMP-1 balance towards proteolytic activity of MMP-9 could be important in MS immune dysregulation [73]. CSF and serum levels of active MMP-9 may indicate a potential biomarker for monitoring MS disease activity, especially because the serum active MMP-9/TIMP-1 ratio is easy to measure. Benesová et al. found significant increases in MMP-9 serum levels and the MMP-9/TIMP-1 ratio in a whole MS group, among patients withRRMS, and in SPMS groups when compared with the healthy controls [74]. They proved that MMP-9 is a useful biological marker in MS as it provides information about the clinical type, disability, and severity of the disease [74]. Moreover, elevated MMP-9 levels have been detected in MS patients’ brain lesions [75,76,77,78], CSF [72,79], and PBMCs [80,81,82], which correlate with disease activity and lesion formation [17,83,84]. In the EAE model, MMP-9 activity was observed at sites of parenchymal border penetration via in situ zymography [85]. Additionally, Pavlovic et al. [86] recently evaluated the utility of CSF biomarkers, such as MMP-9, TIMP-1, the MMP-9/TIMP-1 ratio, and osteopontin (OPN), as indicators of BBB integrity and disease activity in people with relapsing–remitting multiple sclerosis (pwMS), and assessed their changes following autologous hematopoietic stem cell transplantation (aHSCT). Their study indicated that the MMP-9/TIMP-1 ratio and OPN levels were significantly elevated in pwMS compared to those with gadolinium-enhancing lesions or on first-line therapies. Both biomarkers declined significantly after aHSCT and remained low during follow-up. The MMP-9/TIMP-1 ratio showed superior discriminatory capacity and correlated with inflammatory CSF markers. Based on this research, it can be concluded that the CSF MMP-9/TIMP-1 ratio and OPN are elevated in MS and decrease following aHSCT, reflecting reduced inflammation and restored BBB integrity [86]. Based on the above-mentioned studies, it can be concluded that MMP-9 may support disease monitoring and therapeutic evaluation; however, it is not a prospective clinical validation study and does not establish MMP-9/TIMP-1 as a clinical biomarker.

The role of matrix metalloproteinase-2 (MMP-2) in MS centers primarily on chronic neuroinflammation, baseline BBB permeabilization, and long-term tissue remodeling. The dynamics of MS relapses are characterized by the direct stimulation of MMP-9 by pro-inflammatory cytokines, resulting in a rapid surge in its concentration in the CSF and serum, which correlates with new, active demyelinating lesions on MRI and the degradation of the blood–brain barrier. In contrast to MMP-9, MMP-2—another gelatinase—does not react as violently to acute inflammation and is constitutively expressed, acting as a key mediator in the progressive, degenerative stages of the disease. Chronic progression, typical of progressive phenotypes, is characterized by elevated MMP-2 activity and an altered MMP-2/TIMP-2 ratio, serving as a biomarker for insidious neurodegeneration [72]. The study by Avolio et al. has indicated that the MMP-2/TIMP-2 ratio in serum was significantly higher in SPMS and PPMS when compared to short disease duration (SDD) RRMS, but not different from the healthy control group [72]. However, the CSF MMP-2/TIMP-2 ratio did not differ between MS subtypes [72], and the authors did not establish MMP-2 as a causal mediator of progressive neurodegeneration or as a validated biomarker of ongoing neuronal tissue loss. Finally, it was suggested that an increase in the MMP-2/TIMP-2 ratio marks chronic progression in MS [72].

In addition, other authors demonstrated that the MMP-2 expression profile reflects distinct pathophysiological processes separate from the acute inflammatory phase. Benešová et al. revealed a significant elevation in MMP-2 serum levels and in the MMP-2/TIMP-2 ratio in primary progressive and SPMS patient groups when compared with the RRMS group, and this increase was also associated with the degree of disability and disease severity [74]. Their findings proved the clinical utility of MMP-2 as a biomarker for insidious, ongoing loss of nervous tissue [74].

### 3.3. Other Matrix Metalloproteinases (MMPs): MMP-1, MMP-7, MMP-12, MMP-14

Some clinical investigations have reported that other members of the MMP family, such as matrix metalloproteinases-1 (MMP-1) and matrix metalloproteinases-7 (MMP-7), might also be involved in MS pathogenesis. In the study by Camara-Lemarroy et al. [87] on blood levels of cytokines, MMP measurements, serum metabolomics, and immune cell immunophenotyping were performed in participants in the Trial of Minocycline in a CIS. Individuals with early MS/CIS had higher levels of IL-1 receptor antagonist, IL-1β, IL-9, and IP-10 (Interferon-γ-induced protein 10), as well as MMPs 1, 8, and 9 compared with healthy controls. Out of these biomarkers, only levels of MMP-1 differentiated between early MS and CIS and correlated with lesion volume and gadolinium-enhancing lesions on MRI. Moreover, in another study [82] in the Trial of Minocycline in Clinically Isolated Syndrome (MinoCIS), minocycline significantly reduced the risk of conversion to clinically definite multiple sclerosis (CDMS). Moreover, minocycline treatment significantly decreased GFAP levels at month 6 and decreased MMP-7 concentrations at month 1 [88]. Overall, this research indicated that neurofilament light chain (NfL) and GFAP are emerging biomarkers in MS, and that minocycline modulates MMPs.

In a recent investigation, Abed et al. [89] also associated elevated levels of MMPs with MS. Multiple linear regression analyses were conducted to evaluate the independent effects of MS and periodontal disease on biomarker levels. Regression analysis revealed that the presence of MS was significantly associated with elevated serum MMP-9 and MMP-1 and had a significantly greater independent effect on systemic levels of MMP-9 and MMP-1 than periodontal disease [89]. Based on the abovementioned research, MMP-1 may be used as a reliable marker of MS and can differentiate between early MS and CIS. A summary of the main findings for each MMP is presented in Table 2.

Langenfurth et al. indicated that microglial/macrophage matrix metalloproteinases-14 (MMP-14) expression was upregulated in Alzheimer’s disease tissue, in active lesions of multiple sclerosis, and in tissue from stage II stroke as well as in the corresponding mouse models for the human diseases. However, they did not notice upregulation of MMP-14 in microglia/macrophages in the early phase of stroke, in human amyotrophic lateral sclerosis tissue, as well as in human cases of acute brain trauma. These data indicate that MMP-14 expression is not a general marker for activated microglia/macrophages but is upregulated in defined stages of neuroinflammatory and neurodegenerative disorders [90]. Recent studies, however, highlighted the role of intracellular MMPs in mediating either anti- or pro-inflammatory processes [91]. Results of Vos et al. suggest a role for matrix metalloproteinases-12 (MMP-12) during leukocyte migration into MS lesions or during demyelination. In this study, in earlier and later MS lesion stages, MMP-12 immunoreactivity was limited, but in active demyelinating MS lesions, foamy macrophages were MMP-12 positive. Moreover, in active demyelinating lesions, phagocytic macrophages were MMP-12 positive, which might be useful in the treatment strategy for MS [92].

**Table 2:** Summary of the main findings on selected matrix metalloproteinases in multiple sclerosis.

MMP	Study Material/Experimental Model	Main Findings in MS	Neuroinflammatory/Neurodegenerative Effects
MMP-3	Autopsied brain sections from multiple sclerosis cases and controls were screened for the presence of CD20+ B cell aggregates and expression of matrix metalloproteinase-3 [7] Brain slices [8]	Autopsied brain sections from multiple sclerosis patients containing aggregates of B cells expressed a significantly higher amount of matrix metalloproteinase-3 compared to controls [7]. Matrix metalloproteinase-3 dampened the overall activation status of B cells by downregulating CD69, CD80 and CD86 [7]. B cells treated by matrix metalloproteinase-3 produced significantly lower amounts of interleukin-6 [7]. Matrix metalloproteinase-3 altered the proliferation and survival profiles of B cells [7]. CD20 immunostaining intensity was higher in PPMS and SPMS compared with controls. Higher CD20 immunostaining intensity was associated with younger age at onset, SP conversion, wheelchair dependence, and death [8]. MMP-3 triggers microglia to produce proinflammatory and cytotoxic molecules, as well as MMP-3, which in turn contribute to neuronal damage [93]	Decreased overall activation status of B cells [7] Main factor involved in degradation of ECM as well as microglial activation and cytokine release Implicated in neuroinflammation and neurodegeneration Increased these processes [93]
MMP-9	Serum, CSF [65] Brain slices and mouse models [11] CSF samples [80]	The MMP-9/TIMP-1 ratio in serum was higher in SDD RR MS than in PP and in active patients, evaluated either clinically or from magnetic resonance imaging, compared to inactive disease [72] Elevated levels of MMP-9 have been detected in cerebrospinal fluid (CSF), serum, and demyelinating lesions of MS patients, where MMP-9 contributes to myelin breakdown, epitope generation, and leukocyte infiltration [11] MMP-9 did not directly regulate demyelination and remyelination in the CPZ-induced demyelination model [11] The MMP-9/TIMP-1 ratio showed superior discriminatory capacity and correlated with inflammatory CSF markers [86]	In multiple sclerosis (MS), MMP activity in tissues is the result of a balance between MMPs and their tissue inhibitors (TIMPs) [72] MMP-9 increased microglial abundance, highlighting its indirect role in CNS demyelination [11].
MMP-1	Blood samples, serum, immune cells [81] Blood sample, serum [83]	People with early MS/CIS had higher levels of MMP-1 when compared with healthy controls [87] MMP-1 differentiated between early MS and CIS, and was found to correlate with lesion volume and gadolinium-enhancing lesions on MRI [87]	Increased neuroinflammatory effects and the presence of MS were significantly associated with elevated serum MMP-1 [89].
MMP-7	Blood samples [82]	Minocycline treatment significantly decreased MMP-7 concentrations at month 1 [88]	Increased neuroinflammatory effects

## 4. Summary

Multiple sclerosis is a chronic, immune-mediated disorder of the central nervous system characterized by concurrent neuroinflammation, demyelination, and progressive axonal degeneration. The pathophysiology is driven by the infiltration of autoreactive lymphocytes across a disrupted BBB, triggering activation of resident microglia and astrocytes. Upon activation, these parenchymal cells secrete a cascade of pro-inflammatory cytokines and MMPs that degrade the extracellular matrix and amplify tissue injury. Consequently, targeting the interplay between these specific cytokines and MMPs represents a critical therapeutic strategy aimed not only at suppressing acute relapses but, crucially, at arresting long-term neurodegenerative progression.

We report that IL-6 may serve as an indicator of disease activity and reflect the extent of ongoing neuroinflammation. The IL-3–IL-3Rα axis plays an important role in MS pathogenesis by promoting neuroinflammation, while IL-5 is an anti-inflammatory factor. Moreover, IL-9 is a potential prognostic biomarker for monitoring disease progression, highlighting its potential as a therapeutic target in MS, similar to IL-10. In addition, enhancing IL-13 expression may augment the immunomodulatory and neuroprotective effects of stem cell-based therapies for MS. A growing body of evidence proves that selected MMPs also play an important role in the pathogenesis of MS. It was confirmed that MMP-3 is a crucial regulator in neuroinflammation, as well as neurodegeneration, during MS. The dynamics of MS relapses are characterized by the direct stimulation of MMP-9 by pro-inflammatory cytokines, resulting in a rapid surge in its concentration in the CSF and serum, which correlates with new, active demyelinating lesions on MRI and the degradation of the BBB. Thus, MMP-9 might support disease monitoring and therapeutic evaluation; MMP-2 was found to be a differentiation factor between MS phenotypes, and MMP-1 may be viewed as a reliable marker and can differentiate between early MS and CIS. It should be noted, however, that further research based on a prospective clinical-validation study is needed to confirm their role as clinical biomarkers.

With the findings of this review, we show that selected cytokines and matrix metalloproteinases play crucial roles in MS pathogenesis and might be useful in treating patients with multiple sclerosis.

## Figures and Tables

**Table 1:** Summary of the main findings on selected cytokines in multiple sclerosis.

Interleukin	Study Material/Experimental Model	Main Findings in MS	Role in Neuroinflammation and Neurodegeneration
IL-3	CSF	Astrocytes and infiltrating CD4^+^ T cells were identified as the major sources of IL-3 in the inflamed CNS in MS [50]. CSF IL-3 levels were associated with both MS diagnosis and disease severity [50]. The IL-3–IL-3Rα axis contributed to neuroinflammation by mediating communication between immune and glial cells [50].	Neuroinflammation-promoting—IL-3 promotes immune cell recruitment to the CNS through IL-3Rα-expressing myeloid cells [50].
IL-5	CSF, genetic analysis (rs2069812)	The T allele of the IL5 rs2069812 polymorphism was associated with reduced CSF IL-5 levels in RRMS [52]. In progressive MS, the T allele was associated with lower CSF IL-5 concentrations [52].	Potentially anti-inflammatory/protective—lower IL-5 levels were associated with a more pro-inflammatory CSF profile and greater prospective disease activity [52].
IL-6	CSF	Elevated CSF IL-6 levels were associated with progressive MS and correlated with EDSS, MSSS, and GFAP [24]. Detectable IL-6 levels were more frequent in CIS, RRMS, and SPMS than in healthy controls [25]. CSF IL-6 levels positively correlated with contrast-enhancing spinal cord lesions on MRI, indicating ongoing neuroinflammation [27,28]. CSF IL-6 levels were negatively correlated with serum vitamin D levels [28].	Pro-inflammatory—elevated CSF IL-6 levels were associated with ongoing CNS inflammation, greater disease severity, and increased clinical and radiological disease activity [24,25,27,28].
IL-9	CSF, post-mortem brain tissue, model EAE	IL-9 levels were highly expressed in macrophages, microglia, and CD4^+^ T cells, while IL-9R was detected on macrophages/microglia in MS brain tissue [32]. Reduced CSF IL-9 levels were associated with relapses and unfavorable disease course, whereas prednisolone treatment increased CSF IL-9 concentrations [33,34]. In EAE, IL-9 administration reduced clinical disability, neuroinflammation, and synaptic damage, and promoted anti-inflammatory astrocyte polarization through inhibition of NF-κB and GM-CSF expression [35,36].	Anti-inflammatory/neuroprotective—IL-9 reduces macrophage activation and Th17-mediated inflammatory responses and protects against neuroinflammation and synaptic damage [32,33,34,35,36].
IL-10	CSF, serum, model EAE	Higher serum IL-10 levels were associated with lower disability, fewer relapses, shorter disease duration, and better quality of life [41]. CSF IL-10 levels were correlated with EDSS changes during the first year after diagnosis and with the volume of subcortical brain structures (including the caudate nucleus) [38]. In EAE, IL-10 attenuated IL-1β-induced synaptic dysfunction by reducing glutamatergic and enhancing GABAergic neurotransmission, supporting its neuroprotective role [40]. IL-10-related mechanisms have been implicated in EBV-associated MS pathogenesis, while IL-10-producing B10 cells represent a promising immunoregulatory therapeutic strategy [41,42].	Predominantly anti-inflammatory/neuroprotective, but context-dependent—IL-10 regulates inflammatory responses and protects against inflammatory synaptic and neurodegenerative damage; however, its association with MS severity appears to be context-dependent [38,39,40,41].
IL-13	CSF, serum, genetic analysis (rs20541), EAE model	Increased frequency of IL-13-producing CD4^+^ T cells in CSF was observed in RRMS patients during relapse, and these cells positively correlated with EDSS scores [44]. Lower serum IL-13 concentrations were found in RRMS patients compared with controls and were negatively correlated with EDSS scores [46]. The IL13 rs20541 (R130Q) polymorphism was associated with increased MS susceptibility, with A allele carriers showing higher risk compared with GG genotype carriers [47]. In EAE, IL-13-overexpressing hADSCs reduced disease severity and inflammation and promoted remyelination and oligodendrogenesis, indicating potential immunomodulatory and neuroprotective properties [48].	Potentially anti-inflammatory/neuroprotective, but context-dependent—IL-13 showed immunomodulatory and neuroprotective effects in EAE, while increased IL-13-producing T cells were associated with relapse and greater disability in RRMS [44,45,46,47,48].
IL-23 and IL-27	Serum, genetic analysis	The GG genotype of the R381Q polymorphism was associated with higher serum IL-23 concentrations compared with the AG genotype in RRMS patients [53]. A positive correlation between IL-23 concentrations and the duration of disease-modifying therapy (glatiramer acetate or interferon beta) was observed in patients with SPMS [53]. Higher serum IL-27 concentrations were associated with earlier disease onset in RRMS patients, suggesting its potential involvement in the early phase of MS [53].	Unclear—direct effects of IL-23 and IL-27 on neuroinflammation or neurodegeneration were not assessed [53].

## Data Availability

No new data were created or analyzed in this study. Data sharing is not applicable to this article.
